# The effectiveness of ENAR^® ^for the treatment of chronic neck pain in Australian adults: a preliminary single-blind, randomised controlled trial

**DOI:** 10.1186/1746-1340-15-9

**Published:** 2007-07-09

**Authors:** Andrew L Vitiello, Rodney Bonello, Henry Pollard

**Affiliations:** 1 Macquarie Injury Management Group, Department of Health & Chiropractic, Macquarie University, North Ryde, Australia

## Abstract

**Background:**

Current evidence on electrotherapies for the management of chronic neck pain is either lacking or conflicting. New therapeutic devices being introduced to the market should be investigated for their effectiveness and efficacy. The ENAR^® ^(Electro Neuro Adaptive Regulator) therapy device combines Western biofeedback with Eastern energy medicine.

**Methods:**

A small, preliminary randomised and controlled single-blinded trial was conducted on 24 participants (ten males, 14 females) between the ages of 18 to 50 years (median age of 40.5) Consent was obtained and participants were randomly allocated to one of three groups – ENAR, Transcutaneous Electrical Nerve Stimulation (TENS), or control therapy – to test the hypothesis that ENAR therapy would result in superior pain reduction/disability and improvements in neck function compared with TENS or control intervention. The treatment regimen included twelve 15-minute treatment sessions over a six week period, followed by two assessment periods. Visual Analogue Scale (VAS) pain scores, Neck Disability Index (NDI) scores, Patient Specific Functional Scale (PSFS) scores and Short Form 36v1 (SF-36) quality of life scores reported by participants were collected at each of the assessments points throughout the trial (0, 6, 12, 18 and 24 weeks).

**Results:**

Eligible participants (n = 30) were recruited and attended clinic visits for 6 months from the time of randomisation. Final trial sample (n = 24) comprised 9 within the ENAR group, 7 within the TENS group and 8 within the control group. With an overall study power of 0.92, the ENAR group showed a decrease in mean pain score from measurement at time zero (5.0 ± 0.79 95%CI) to the first follow-up measurement at six weeks (1.4 ± 0.83 95%CI). Improvement was maintained until week 24 (1.75 ± 0.9 95%CI). The TENS and control groups showed consistent pain levels throughout the trial (3.4 ± 0.96 95%CI and 4.1 ± 0.9 95%CI respectively). Wald analysis for pain intensity was significant for the ENAR group (p = 0.01). Six month NDI scores showed the disability level of the ENAR group (11.3 ± 4.5 95%CI) was approximately half that of either the TENS (22.9 ± 4.8 95%CI) or the control (29.4 ± 4.5 95%CI) groups. NDI analysis using the Wald method, indicated significant reductions in disability only for the ENAR group (p = 0.022). PSFS results also demonstrated significantly better performance of ENAR (p = 0.001) compared to both alternative interventions. Differential means analysis of the SF-36 results favoured ENAR for all of the subscales. Six of the initial 30 participants discontinued the trial protocol.

**Conclusion:**

ENAR therapy participants reported a significant reduction in the intensity of neck pain (VAS) and disability (NDI), as well as a significant increased function (PSFS) and overall quality of life (SF-36) than TENS or control intervention participants. Due to the modest sample size and restricted cohort characteristics, future larger and more comprehensive trials are required to better evaluate the potential efficacy of the ENAR device in a more widely distributed sample population.

**Trial Registration:**

This study has been registered with the Australian Clinical Trials Registry (ACTR): ACTRN012606000438550.

## Background

The overall prevalence of neck pain is approximately 60% and point prevalence has been estimated to be between 14% and 18% [1-3]. Chronic neck pain is similar to chronic low back pain in that it is difficult to diagnose, resistant to common therapeutic interventions, and is a financial burden on society. A recent Cochrane review found that "The current evidence on Galvanic current (direct or pulsed), iontophoresis, Transcutaneous Electrical Nerve Stimulation (TENS), Electronic Muscle Stimulation (EMS), Pulsed Electromagnetic Field (PEMF) and permanent magnets is either lacking, limited, or conflicting" [4]. Despite the paucity of evidence, electrotherapy remains a popular treatment modality with new devices periodically introduced to the market. These products are largely untested in terms of properly conducted randomised controlled trials.

The Electro Neuro Adaptive Regulator (ENAR) therapy device is a hand held, battery powered, electrotherapy device which, although gaining popularity, is untested and unreported for its clinical efficacy. The manufacturer claims the device was developed by a team of Russian doctors and scientists at Sochi University in the late 1970s, the device combines Western electrical biofeedback with Eastern energy medicine.

This preliminary study investigated the response of people with non-complicated chronic neck pain (NCCNP) to two treatments – ENAR and TENS – and one control procedure. NCCNP was defined as neck pain of duration of three months or longer without referral of pain in the upper limbs or hand paraesthesia [5]. The aim of the project was to evaluate if the application of ENAR therapy resulted in statistically significant changes in the measured outcomes when compared to the use of TENS or a control therapy. As outcomes data on the ENAR device had not been reported in the literature this study formed a foundation for knowledge relating to the use of this type of therapy. A methodology was developed which allowed the assessment of both the short and medium term effects of ENAR on this sample of people with neck pain.

### Aim of this study

The aim of this study was to test a recently released and largely untested electro-physical therapy (ENAR) in reducing the symptoms of non complicated chronic neck pain (NCCNP). In order to facilitate this aim the researchers collected participant reported, paper based outcome measure data from a randomly allocated cohort (n = 24) of chronic neck pain sufferers, including neck pain intensity, neck function, disability and its overall influence on quality of life.

## Methods

A randomised, controlled single-blinded preliminary study was developed for application in a university clinical teaching setting. Print media advertising in Sydney, Australia was used from October-November 2003 and, following application of the inclusion/exclusion criteria, 30 participants were randomly allocated to one of three groups: ENAR therapy, TENS therapy and control. Informed consent was obtained and stratified randomisation was conducted using a freely available and peer reviewed design method known as urn randomisation [6]. The subject allocation was concealed from all persons who had a potential to interact with the trial participants. Additional File 1 outlines the flow of participants throughout the trial, including the intervals between outcomes assessments and the frequency of each treatment session for each group as set out by the CONSORT guidelines(Additional file 2) [7]. A six-week schedule of treatment was administered by trained final year chiropractic interns at the respective treatment/university outpatient clinics, to the neck and upper back of participants. ENAR treatment involved setting the device to an intensity determined by the individual participant's tolerance and applying it to the skin in accordance with the directions set out in the manufacturer's product literature. It was applied with light pressure to the posterior neck and upper thorax in gentle stroking movements. TENS was utilised in this study due to the similarity of its sensory stimulus compared to the ENAR device. Its dimensions, application to skin regions, and overall potential treatment sensations were of a similar nature, thereby facilitating a blinded comparison. For TENS therapy, electrodes were applied to the skin overlying the posterior surface of the neck and upper thorax regions, matching the area stimulated by the ENAR. Dosage was set to comfortable tolerance level set below muscle fasciculation response. For the control group, the ENAR device was used and applied in an identical manner to the ENAR therapy group except that the unit was turned on and then immediately off before being applied to the skin. Prior to commencement of the study all participants were advised of the potential they may not feel the electrotherapy during the trial, thereby minimising any perception of a treatment either occurring or not. Each of the groups received 10 minutes of their respective therapy, including the control group according to the schedule outlined in Additional File 1. Visual Analogue Scale (VAS) pain scores [8], Neck Disability Index (NDI) scores [9], Patient Specific Functional Scale (PSFS) scores [10] and Medical Outcomes Study 36-Item Health Survey Version 1 (^© ^1988, 2002 Medical Outcomes Trust & QualityMetric Incorporated) (SF-36) [11] scores reported by participants were collected during weeks 1, 6, 12, 18 and 24 of the trial. Each of these indices were measured both immediately before and after the treatment administration period (weeks 1 and 6) and on each six weekly intervals until 24 weeks had elapsed (weeks 12, 18 and 24). This enabled measurement of the short and medium term effects of the therapy [5]. A separate group of assessment administrators was created. The administrators were blinded as to which intervention each participant received and were only concerned with ensuring all questionnaires were filled correctly and tabulating data for independent analysis. This was designed to address the potential for participants becoming unblinded as to the type of therapy being delivered.

The Human Ethics Committee of Macquarie University approved this study prior to commencement. Further, the study design complies with Australian National Health and Medical Research Council's National Statement on Ethical Conduct in Research Involving Humans [12] and with the Helsinki Declaration [13] on Human Research.

Of the 30 participants consenting to participate in the trial, 24 continued to the end of the phase 1 stage of the study. This equated to an overall participation rate of 80%. For group allocation, all subjects were randomised using the Urn Randomising Software^® ^program (Stout 1994- Project Match- University of Connecticut Health Centre) and each subject was allocated a Trial Participant Number (TPN) to allow blinding during the clinical trial.

Data were tabulated and statistically analysed using GenStat^® ^(v 9.0) 2006 and SPSS^® ^(v13) 2005 software packages. (REML) Linear Mixed Model analysis (with repeated measures) was performed on pain, disability and functional parameters (CI = 95%) with a Logistic Regression Analysis (Wald Test for fixed effects) being performed in order to determine statistical significance between groups. Descriptive and interferential methods were applied to the data using a standard significance level (alpha) of 0.05 where applicable. SF-36 data were compiled using the Clinical Outcomes Evaluation System v3.74 (COES^®^) – Repatriation Hospital 1996 using standard scoring algorithms and analysis of means and standard deviation in that program.

## Results

**Figure 1:**
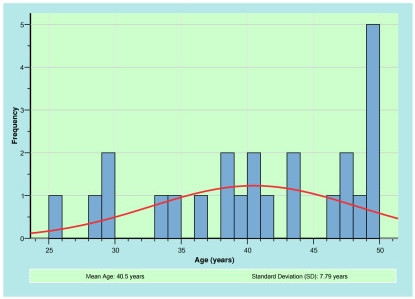
Frequency analysis of participant age.

Ten males and 14 females completed the trial. The racial distribution of the cohort is consistent with that reported in the Australian 2001 Census data of people living in the same geographical area (Ryde, New South Wales) [14]. Table 2 demonstrates the breakdown of participant race within the project cohort, Table 3 identifies the allocation of participants to the individual treatment groups. Figure 1 graphically represents the age distribution of the cohort with a median age of 40 years.

**Table 2:** Frequency analysis of participant race

		Frequency	Percent	Valid Percent	Percent
Valid	asian	5	20.8	20.8	20.8
	caucasian	19	79.2	79.2	100.0
	**Total**	**24**	**100.0**	**100.0**	

**Table 3:** Frequency analysis of participant allocation to each treatment group

**Treatment Groups**					
		Frequency	Percent	Valid Percent	Cumulative Percent

Valid	ENAR	9	37.5	37.5	37.5
	TENS	7	29.2	29.2	66.7
	SHAM	8	33.3	33.3	100.0
	**Total**	**24**	**100.0**	**100.0**	

**Figure 2:**
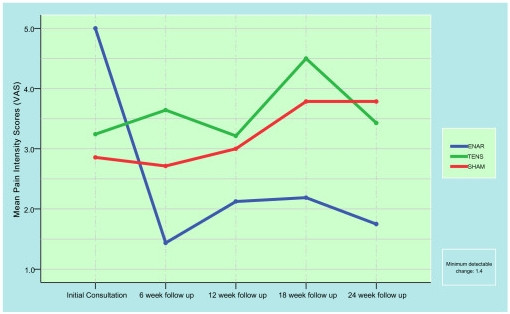
Mean pain intensity (VAS) scores for each intervention.

Figure 2 is a graphic representation of mean pain intensity scores (VAS) for each intervention group across trial and follow-up timeframes. The ENAR group presents a decrease in mean pain scores from the initial measurement at time zero to the first follow-up measurement at six weeks. This improvement is maintained throughout the 24 week follow-up period. The TENS and control treatment groups each maintained consistent pain levels throughout the trial. The ENAR therapy was successful in significantly reducing chronic neck pain intensity (p = 0.01) from 5.0 ± 0.79 at baseline to 1.75 ± 0.90 at the 24 week follow-up. The TENS or control groups had no such reductions in pain intensity.

Table 6 indicates means and standard errors of (NDI) as a function of intervention type. Smaller numbers indicate less disability/improved function. The ENAR therapy group commenced the trial with a similar baseline disability status as the control group, but, on average, both these groups were more disabled (higher score) than the TENS group at the commencement of the trial. By the conclusion of the trial 24 weeks later, the disability score of the ENAR group was approximately half that of either the TENS group or the control group.

**Table 6:** NDI means and standard errors as a function of intervention

				95% Confidence Interval	
				
Intervention	Assessment	Mean	Std. Error	Lower Bound	Upper Bound
ENAR	Initial consultation	30.750	3.001	24.490	37.010
	6 week follow-up	19.113	3.448	11.920	26.305
	12 week follow-up	17.250	4.303	8.275	26.225
	18 week follow-up	15.000	3.857	6.955	23.045
	24 week follow-up	11.250	4.511	1.840	20.660

TENS	Initial consultation	20.286	3.208	13.594	26.978
	6 week follow-up	19.714	3.686	12.025	27.404
	12 week follow-up	21.143	4.600	11.548	30.738
	18 week follow-up	26.571	4.123	17.971	35.172
	24 week follow-up	22.857	4.823	12.797	32.917

SHAM	Initial consultation	30.500	3.001	24.240	36.760
	6 week follow-up	21.500	3.448	14.307	28.693
	12 week follow-up	27.625	4.303	18.650	36.600
	18 week follow-up	26.500	3.857	18.455	34.545
	24 week follow-up	29.375	4.511	19.965	38.785

**Figure 3:**
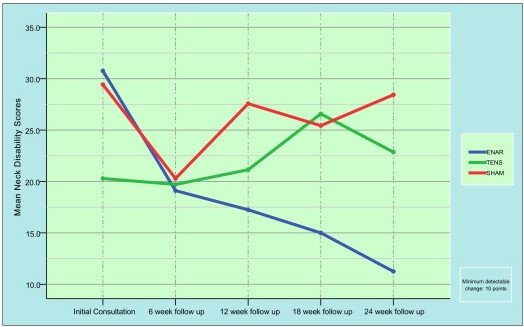
Mean Neck Disability Scores (NDI) for each intervention.

Figure 3 is a graphic presentation of the NDI results. All participants were chronic pain sufferers as determined by the inclusion criteria. No subject displayed the severe or complete levels of disability usually associated with acute injury. Similar results for neck disability were obtained, showing the TENS and control groups enjoyed no real benefit from their intervention whilst the ENAR group reduced their disability index score to less than half of their initial values during the 24 week trial. In clinical terms this equates to a change from borderline 'Moderate/Mild Disability' to borderline 'Mild/No Disability' status.

Participants receiving ENAR therapy had statistically significant reductions in NDI scores (p = 0.02). These scores reduced from 30.8 ± 3 at the beginning of the trial to 11.3 ± 4.5 at the 24 week follow-up stage. Over the same 24 week period, participants undergoing either the TENS or control therapy did not display any statistically significant reductions in their NDI scores (TENS from 20.3 ± 3.2 to 22.9 ± 4.8, and control from 30.5 ± 3.0 to 29.4 ± 4.5.).

The minimum clinically detectable change in a participant's presenting symptoms is 10 NDI points, or 10% [9]. As the results indicate, participants receiving either the TENS or control treatments did not present any clinically visible improvements after 24 weeks. This is highlighted by neither of the two groups progressing from the initial classification of disability throughout the 24 week trial period. Which is in contrast to the ENAR group, who over the same 24 week period had both clinically significant changes in their NDI scores and were also significantly closer to being classified as having 'No Disability' according to the NDI scoring schedule.

Table 8 presents means and standard errors of neck functioning (PSFS) relating to the intervention type. In this analysis larger scores depict higher levels of function or functional improvement. The ENAR groups more than doubled its functional assessment scores, unlike the TENS group (which resulted in virtually no change in level of function), and the control group (which resulted some loss of function).

**Table 8:** PSFS means and standard errors as a function of intervention

				95% Confidence Interval	
				
Intervention	Assessment	Mean	Std. Error	Lower Bound	Upper Bound
ENAR	Initial consultation	3.566	.699	2.102	5.030
	6 week follow-up	7.712	.848	5.937	9.488
	12 week follow-up	7.762	.913	5.853	9.672
	18 week follow-up	8.025	.911	6.118	9.932
	24 week follow-up	8.475	.943	6.502	10.448

TENS	Initial consultation	5.371	.748	3.806	6.936
	6 week follow-up	6.157	.907	4.259	8.055
	12 week follow-up	5.586	.976	3.544	7.627
	18 week follow-up	5.014	.974	2.975	7.053
	24 week follow-up	5.614	1.008	3.505	7.723

SHAM	Initial consultation	6.529	.748	4.964	8.094
	6 week follow-up	6.657	.907	4.759	8.555
	12 week follow-up	5.443	.976	3.401	7.485
	18 week follow-up	6.757	.974	4.718	8.796
	24 week follow-up	5.957	1.008	3.848	8.066

**Figure 4:**
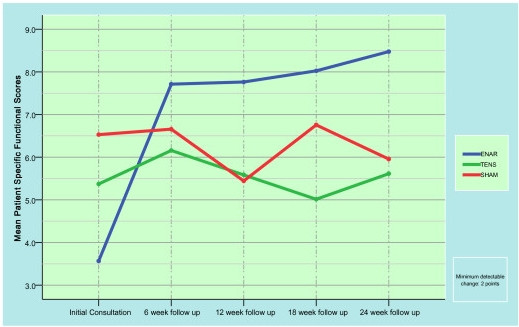
Mean and SE of Patient Specific Functional Scale (PSFS) scores.

Figure 4 graphically demonstrates the changes in specific neck function (PSFS) which occurred with each treatment group over the 24 week trial period. Of particular note is the statistically significant improvement in overall neck function in the ENAR group from the beginning of the trial (3.6 ± .07) until immediately after cessation of treatment (7.7 ± 0.8). These scores continued to steadily increase during weeks 12 (7.8 ± 0.9), 18 (8.0 ± 0.9) and 24 (8.5 ± 0.9) where significant differences were still evident (p = 0.001). Participants undergoing either TENS or control treatments exhibited PSFS scores at week 1(5.5 ± 0.7 and 6.5 ± 07) that did not significantly change at either the 6 week(6.2 ± 0.9 and 6.7 ± 0.9), 12 week(5.6 ± 1.0 and 5.4 ± 1.0), 18 week(5.0 ± 10. and 6.8 ± 1.0) or 24 week(5.6 ± 1.0 and 6.0 ± 1.0) stages of the trial.

If the minimally clinical detectable change in PSFS score is 2, that is, a 20% change in the overall PSFS score, [15] a significant clinical advantage was achieved with the ENAR treatment over the entire 24 weeks compared to both the TENS and control therapy options. While the ENAR therapy was successful in improving the participant's neck function scores by 136% and exhibiting clear clinical changes, the changes for the TENS and control therapies were 3.7% and -7.7% respectively for the same 24 week period.

Analysis of Quality Of Life (QOL) factors was undertaken using the SF-36 [16]. The analysis presented was graphically compiled by the COES^® ^(Clinical Outcomes Evaluation Software) package developed by the Repatriation General Hospital's Orthopaedic Unit in association with the Information Management Division, South Australian Health Commission (SAHC). No clinical advantage was noted in any of the eight key indices in either the TENS or control therapy groups over the 24 week period. By contrast, participants receiving the ENAR therapy had significant clinical improvements in all eight subscales of the SF-36 questionnaire over the same 24 week period.

The QOL SF-36 data indicates the ENAR treatment participants scored better than their TENS and control counterparts on each of the eight measured subscales. Of particular note, the ENAR group equalled or outperformed the 1995 ABS NHS normative values particularly when comparing the 'Role Emotional' and 'Social Functioning' psychometric parameters.

The reduction in the mean VAS scores is an indicator of success when compared to the lack of VAS reduction in the TENS or control therapy. The ongoing reduction of the VAS score beyond the 6 week period is a clinically useful finding in this chronic pain cohort.

To better understand the dose response characteristics of ENAR more frequent measurements of pain, treatment times and treatment frequency should be investigated. It is unknown whether equal success could be gained over a lesser treatment period. Conversely, a longer treatment regimen of eight or ten weeks may generate greater or longer lasting treatment effects and lead to further pain reduction. The potential value of follow-up periods of therapy was not investigated during this trial though it was interesting to find significant short/medium term pain reduction and improvements in other outcomes measures.

Such conjecture will remain unanswered until further trials are conducted using varying treatment variables. A follow-up trial, with participants given ongoing or subsequent ENAR treatment blocks, may uncover further effects of any additional treatment sessions. Although rarely performed in similar neck pain research, such studies can be important in determining the ultimate value of a therapy in chronic conditions unlikely to resolve in the short term and under what treatment schedule it is best applied.

The strength of the results were somewhat weakened due to the influence of larger variations in scores associated with the small sample sizes during weeks 12–24. Despite this, the reductions in mean VAS values in the ENAR group were maintained.

In assessing any therapy whilst obtaining a statistically significant result is important, a far more relevant indicator of ultimate usefulness is the determination of clinical significance. A clinically significant decrease in pain levels has been quantified a 36% reduction in a VAS scale [17]. In a clinical setting this equates to a reduction of 24 mm along a 100 mm scale. The participants receiving the ENAR therapy had demonstrable reductions in pain intensity immediately after the six week treatment period (from VAS 5.0 to 1.4 equalling a 72% reduction). This effect continued with reductions in measured outcomes after 24 weeks when compared to their initial presentation (from 5.0 to 1.8 equalling a 64% reduction). Other authors find different levels of change to be clinically relevant. Gallagher concludes a 13 mm difference on the VAS represents the smallest measurable change in pain severity that is clinically important [18]. Dickson [19] reports a 13 mm VAS change (i.e. at least 38%) represents a clinically significant change in pain when the initial VAS score is less than 34 mm. However, among participants with initial VAS scores of at least 67 mm, a 28 mm change (i.e. 42%) is required to represent a clinically significant change in pain. Ideally, the results of this study should be reproduced with longer term follow-up and a greater number of participants to determine the length of the treatment effect in the absence of ongoing treatment.

Reduction in VAS scores were not observed in participants receiving either the TENS or the control therapy over the 24 week trial period.

The benefit of this program is its standardisation and simplification of overall administration and scoring of the Quality of Life (QOL) SF-36 questionnaire's eight key components – physical functioning (PF), role physical (RP), bodily pain (BP), general health (GH), vitality (VT), social functioning (SF), role emotional (RE) and mental health (MH) – and its comparison of results to average Australian values collected during the 1995 Australian Bureau of Statistics National Health Survey (1995 ABS NHS) [20]. With respect to the relative impact of ENAR treatment on QOL SF-36 indicators, participants receiving the ENAR therapy displayed significant clinical improvements in all aspects of the SF-36 questionnaire. These improvements were so considerable that the 'Role Emotional' and 'Mental Health' components of the ENAR cohort outscored the 1995 Australian population norms. However, considering the relatively small sample size, care must be exercised when comparing the results with large non-specific population datasets such as the Australian NHS data. This is a significant achievement in a population of people with chronic pain and perhaps serves to reinforce that chronic pain syndromes have strong psychosocial impact and such impact may also be affected by reduced pain.

### Limitations of this study

The study has limitations. The first limitation is the modest sample size of 24 participants. Despite this, the authors present real and statistically significant positive trends relating to the ENAR therapy in treating people with NCCNP. As this was a preliminary study discussion of these results in a wider context of NCCP should be limited to the focused sample size investigated.

A second limitation of this study was its lack of blinding of the practitioners delivering the therapies. This was due to the inability of providing a device, in this case a TENS unit, that was physically identical to the ENAR device in order that the therapy administrators could be blinded as to the exact intervention being given to the participant. The authors determined an adequate solution to this limitation would be to provide a separate 'assessment administrator' who would be blinded as to the intervention given to the participant and only gather outcomes based information on weeks 1, 6, 12, 18 and 24 as well as tabulating data for independent statistical analysis. Future studies should be directed towards providing a device that could function as both a TENS and ENAR.

Thirdly, researchers who embark on prospective clinical trials are acutely aware of the randomisation tool to maintain groups with similar baseline characteristics. Using human participants, who for any number of reasons may withdraw from a study, make the maintenance of "matched" groups a tenuous goal. In this study, there were differences between groups for the variable of race. However, there is neither recent nor significant evidence supporting the premise that there may be significant differences in NCCNP rates between different sub-populations (Anglo-Saxon, Australian Aboriginal, Asian, African, American, etc). The authors suggest the sample groups after the participant drop-out, although somewhat different at baseline, were sufficiently homogeneous with regard to the racial breakdown (Asian vs Anglosaxon) to be regarded as equal. Age, sex, and chronicity of study participants were not martched in this study and should be addressed in greater detail in future studies.

Fourthly, the authors acknowledge the more stringent intention to treat (ITT) analysis was not performed on this cohort and consider it a limitation of the study. 24 of the 30 initial participants that consented to participate in the trial, actually completed the entire protocol. Those who discontinued included one from the ENAR treatment group who could not maintain the regular treatment schedule determined by the testing protocol. From the TENS group one participant discontinued due to unexpected time scheduling constraints while two others due to a worsening of their neck pain symptoms. Within the control group, two participants failed to continue due to a worsening of their neck pain symptoms. No other adverse reactions were reported by the participants as a result of either the ENAR or TENS interventions during the trial period. Due to the premature departure of six participants data for an ITT analysis could not be gathered, resulting in all data being analysed on a *per protocol *basis [21]. In future studies an early data collection point, perhaps 2 or 3 weeks in to the 6 week regime could be used to establish trend with an ITT analysis. However, because withdrawals from the TENS and Control groups were mostly due to worsening symptoms, it is likely that an ITT analysis would have further favoured the ENAR intervention.

Much of the treatment rendered by practitioners for neck pain is pharmacologic, manual, exercise of multimodal in nature [22]. Further studies should consider the role of ENAR as an alternative or in combination with these more traditional therapies.

## Conclusion

In this small trial, participants who received ENAR therapy experienced greater reductions in the intensity of neck pain and disability, and increased function and overall quality of life, compared with participants receiving either TENS therapy or placebo electrotherapy.

## Competing interests

The authors and their employing institution have no commercial stake in the research or its outcomes other than being recipients of the research grant to perform the investigation. They did not receive any direct financial benefits from the potential success or otherwise that may result from the use or marketing of the therapy in question.

## Authors' contributions

ALV conceived the study, participated in its design, collected data and co-ordinated and helped draft the manuscript.

RB participated in its design, collected data and co-ordinated and helped draft the manuscript.

HP participated in its design and co-ordinated and helped draft the manuscript.

Each of the authors read and approved the final manuscript.

**Figure 5:**
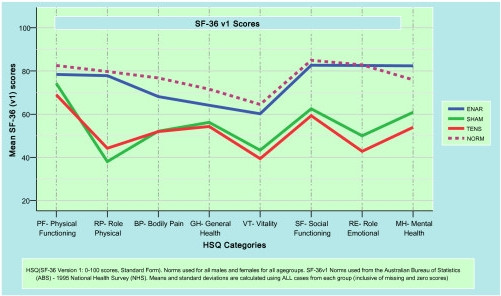
Mean SF-36 scores and national norms.

**Table 1:** Inclusion and Exclusion criteria for participants

**Inclusions**
Adults from the geographically local population surrounding Sydney, Australia.
Aged between 18 and 50 years.
Chronic neck pain of a minimum 6 weeks duration.
Assessed as non-complicated neck pain, i.e. no sign or symptom implying cervical spine discogenic disease or radiculopathy.
**Exclusions**
Suspicion of relevant Red Flag Conditions such as; Spinal fractures, Osseous and Cartilaginous infections, Inflammatory Arthritic conditions, and Malignancy.
Yellow Flag Conditions such as; Non-finalised Workers Compensation or Third Party Insurance Claim, Any other non-finalised compensatory litigation.
WAD grade 1–4 whiplash injury within the last six months.
Presence of significant vascular disease.
Severe or acute relapse of neck pain within the last three months.
Motor vehicle accident, serious falls or any other accident requiring medical/hospital treatment within the last three months.
Current neurological signs, symptoms or syndromes, e.g. muscle wasting or nerve root signs, epilepsy or paraplegia.
Pregnancy or likelihood of pregnancy within the trial period.
Spinal or orthopaedic surgery within the past two years.
Bowel, or bladder/sexual dysfunction as a result of either lumbar spine or prostate dysfunction
Currently undergoing a course of manual therapy or psychological intervention.
Participants not prepared to attend 12 treatment sessions within the first six weeks and a further three assessment sessions over the next 18 weeks.

**Table 4:** VAS (Pain) means and standard errors as a function of intervention

				95% Confidence Interval	
				
Intervention	Assessment	Mean	Std. Error	Lower Bound	Upper Bound
ENAR	Initial consultation	5.000	.789	3.354	6.646
	6 week follow-up	1.438	.829	-.293	3.168
	12 week follow-up	2.125	.981	.078	4.172
	18 week follow-up	2.188	.813	.492	3.883
	24 week follow-up	1.750	.896	-.119	3.619

TENS	Initial consultation	3.243	.844	1.483	5.003
	6 week follow-up	3.643	.887	1.793	5.493
	12 week follow-up	3.214	1.049	1.026	5.402
	18 week follow-up	4.500	.869	2.688	6.312
	24 week follow-up	3.429	.958	1.431	5.427

SHAM	Initial consultation	3.250	.789	1.604	4.896
	6 week follow-up	3.250	.829	1.520	4.980
	12 week follow-up	3.438	.981	1.391	5.484
	18 week follow-up	4.063	.813	2.367	5.758
	24 week follow-up	4.063	.896	2.194	5.931

**Table 5:** Wald statistic for VAS (Pain)

**Fixed term**	**Wald statistic**	**d.f.**	**Wald/d.f.**	**P**
Time	10.39	4	2.60	0.034
RxGroup	1.34	2	0.67	0.512
Time_RxGroup	20.07	8	2.51	0.010*

**Table 7:** Wald statistic for NDI

**Fixed term**	**Wald statistic**	**d.f.**	**Wald/d.f.**	**P**
Time	14.50	4	3.63	0.006
RxGroup	5.23	2	2.62	0.073
Time_RxGroup	17.93	8	2.24	0.022*

**Table 9:** Wald Statistic for PSFS

**Fixed term**	**Wald statistic**	**d.f.**	**Wald/d.f.**	**P**
Time	23.20	4	5.80	<0.001
RxGroup	1.89	2	0.95	0.388
Time_RxGroup	34.25	8	4.28	<0.001*

## Supplementary Material

Additional file 1 Study Protocol. Figure showing the flow of three participant groups through the trial. Click here for file

Additional file 2 The Consort Flowchart. This figure depicts the standardised protocol showing enrolment, allocation, follow-up and analysis stages of the trial. Click here for file
