# Supplementary material for: The effectiveness of ENAR® for the treatment of chronic neck pain in Australian adults: a preliminary single-blind, randomised controlled trial
Source: Chiropr Osteopat. 2007 Jul 9;15:9. doi: 10.1186/1746-1340-15-9 (PMC1963325; doi:10.1186/1746-1340-15-9)
Supplement: Additional file 2 — The Consort Flowchart. This figure depicts the standardised protocol showing enrolment, allocation, follow-up and analysis stages of the trial. [file 1746-1340-15-9-S2.doc]

## **ADDITIONAL FILE 1- CONSORT FLOWCHART**
