# Supplementary material for: The effectiveness of ENAR® for the treatment of chronic neck pain in Australian adults: a preliminary single-blind, randomised controlled trial
Source: Chiropr Osteopat. 2007 Jul 9;15:9. doi: 10.1186/1746-1340-15-9 (PMC1963325; doi:10.1186/1746-1340-15-9)
Supplement: Supplementary file 2 [file 1746-1340-15-9-S1.doc]

ENAR Pilot Study Protocol

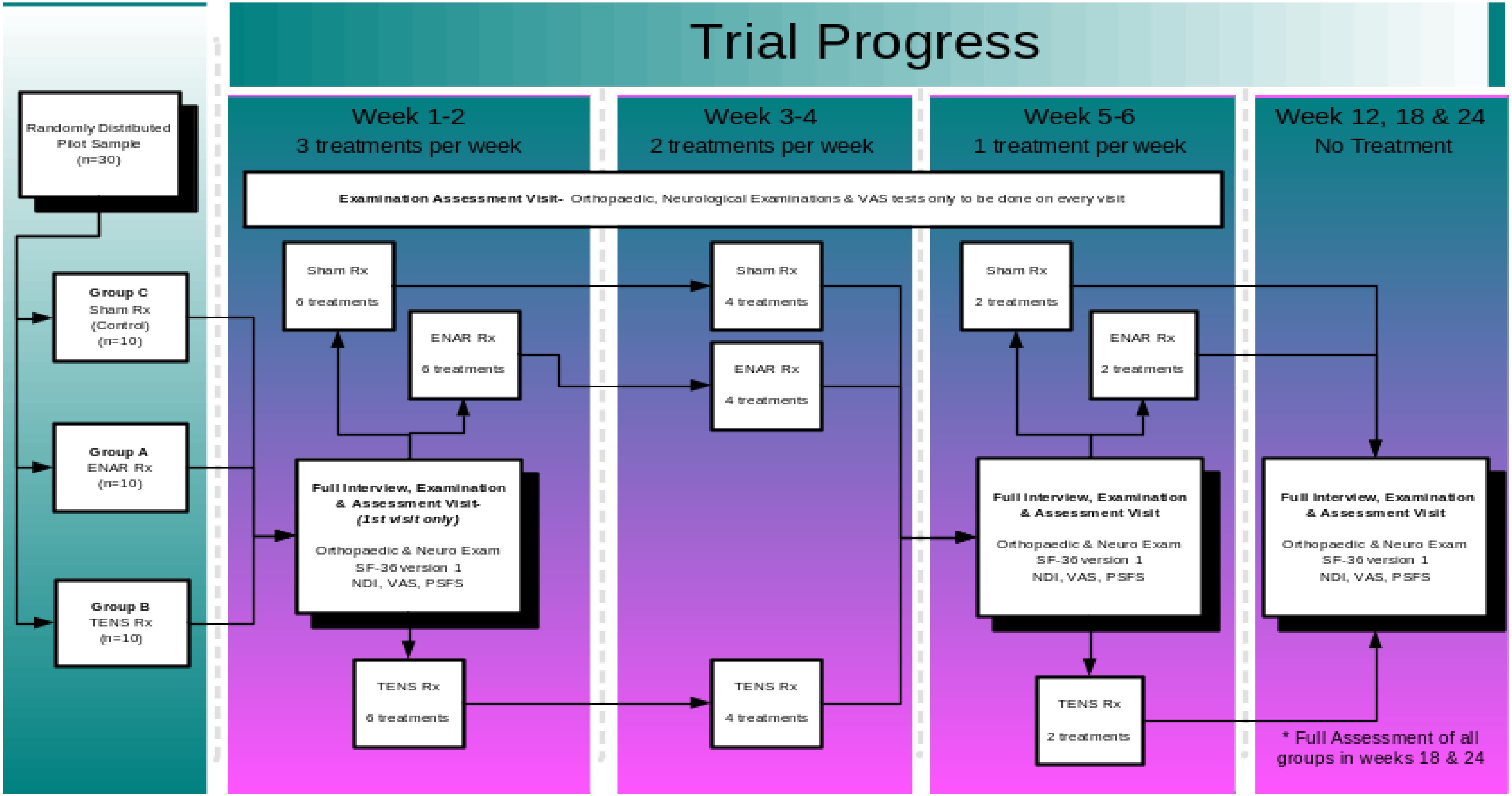

Treatment: Phase 1. 5 min at site of pain either side of spine
Phase 2. 2 min at each "Sticky Point" at either side of spine (max of five)
